# Financial Toxicity and Follow‐Up Quality of Life Among Breast Cancer Survivors in the BOLD Cohort

**DOI:** 10.1002/pon.70630

**Published:** 2026-10-14

**Authors:** Ivan H. C. Wu, Rachel I. Vogel, Patricia I. Jewett, Anne H. Blaes

**Affiliations:** ^1^ Division of Epidemiology & Community Health School of Public Health University of Minnesota Minneapolis Minnesota USA; ^2^ Masonic Cancer Center University of Minnesota Minneapolis Minnesota USA; ^3^ Department of Obstetrics, Gynecology, and Women's Health Division of Gynecologic Oncology University of Minnesota Medical School Minneapolis Minnesota USA; ^4^ Division of Environmental Health Sciences School of Public Health University of Minnesota Minneapolis Minnesota USA; ^5^ Division of Hematology, Oncology, and Transplantation Department of Medicine University of Minnesota Medical School Minneapolis Minnesota USA

**Keywords:** breast cancer survivorship, FACT‐G, financial toxicity, patient‐reported outcomes, quality of life

## Abstract

**Background:**

Financial toxicity is prevalent in cancer survivorship and has been associated with poorer health‐related quality of life (HRQOL), yet longitudinal evidence remains limited. We examined associations between financial toxicity and HRQOL at baseline and 4‐month follow‐up among breast cancer survivors in the BOLD cohort.

**Methods:**

Data were drawn from self‐report questionnaires completed by 182 breast cancer survivors at baseline, of whom 146 completed 4‐month follow‐up surveys. Financial toxicity was assessed at baseline using the Comprehensive Score for Financial Toxicity (COST). HRQOL outcomes were assessed at baseline and approximately 4 months later using the Functional Assessment of Cancer Therapy–General (FACT‐G) subscales and total score, and a single‐item measure of sleep disturbance. Multivariable linear regression models estimated adjusted cross‐sectional associations and longitudinal associations between baseline financial toxicity and follow‐up outcomes, adjusting for baseline levels of the corresponding outcome. Longitudinal measurement invariance of the COST was evaluated.

**Results:**

Greater baseline financial toxicity was associated with lower baseline physical, social/family, emotional, and functional well‐being, lower FACT‐G total score, and greater sleep disturbance. In longitudinal models, higher baseline financial toxicity was associated with lower follow‐up physical, social/family, emotional, and functional well‐being and lower FACT‐G total score after adjustment for demographic and clinical covariates and the corresponding baseline outcome. Baseline financial toxicity was not associated with follow‐up sleep disturbance.

**Conclusions:**

Financial toxicity was associated with poorer HRQOL at baseline and with lower physical, social/family, emotional, functional, and overall HRQOL at follow‐up. These findings support greater attention to financial toxicity in survivorship care.

## 1 Introduction

Cancer survivorship has expanded substantially in recent years, accompanied by increased attention to survivors' long‐term well‐being [1]. A growing concern is the economic burden and distress associated with cancer care costs also known as *financial toxicity* [2]. Survivors often face substantial out‐of‐pocket expenses and income loss that may persist years after treatment, reflecting ongoing care needs and late cancer effects [1, 3]. Evidence suggests that while many cancer survivors experience objective financial hardship, many more report subjective financial distress [2] highlighting how financial toxicity represents a persistent challenge for a large segment of the survivorship population.

Financial toxicity is conceptualized as a multidimensional construct encompassing material hardship, psychological distress related to financial strain, and cost‐related behavioral responses [4, 5]. Cancer‐related financial hardship disproportionately affects socially and economically vulnerable groups, including women, younger survivors, individuals with lower income, and those without adequate insurance coverage [2]. Among women with breast cancer, treatment‐related costs can consume a significant portion of household income, placing them at risk for financial toxicity and worsened health outcomes due, in part, to challenged self‐management of chronic illnesses (e.g., medication nonadherence and delayed or forgone care) [2]. Financial toxicity has been consistently associated with poorer health‐related quality of life (HRQOL), heightened psychological distress, and difficulty meeting basic needs [1, 6].

Much of the evidence linking financial toxicity to patient‐reported outcomes in the US remains cross‐sectional [7] with fewer studies examining whether financial toxicity is associated with subsequent differences in quality of life over time. Available longitudinal studies yield mixed evidence regarding which quality‐of‐life domains are prospectively associated with financial toxicity [8, 9, 10], and prospective evidence remains particularly limited among US‐based breast cancer survivors. To address these gaps, the current study used longitudinal data to examine associations between baseline financial toxicity and HRQOL at baseline and over short‐term follow‐up among breast cancer survivors. We hypothesized that greater baseline financial toxicity would be associated with lower baseline HRQOL across FACT‐G domains and greater sleep disturbance. We further hypothesized that greater baseline financial toxicity would be associated with lower HRQOL at follow‐up after adjustment for baseline outcome levels.

## 2 Methods

### 2.1 Study Design and Participants

We conducted a secondary analysis of data from the *Breast Oncology—Life after Diagnosis (BOLD)* cohort study of breast cancer survivors receiving oncology care at the University of Minnesota. Methods have been described elsewhere [11]. Briefly, individuals with a previous diagnosis of breast cancer who were ≥ 18 years and English‐speaking were recruited between 2018 and 2021. The BOLD study purposely oversampled younger patients and individuals with metastatic breast cancer, both groups of which are often small in mixed breast cancer cohorts. Individuals were recruited in‐person and by mail and were eligible regardless of time since initial diagnosis. Participants completed baseline and follow‐up surveys online (REDCap) or via paper every 4 months for up to 2 years. This analysis includes participants who completed baseline (T1) and 4‐month (T2) follow‐up patient‐reported outcomes assessments. This cohort study was approved by the University of Minnesota Institutional Review Board (BOLD: STUDY00002747) and all participants provided written informed consent.

### 2.2 Measures

#### 2.2.1 Financial Toxicity

Financial toxicity was measured at baseline using the Comprehensive Score for Financial Toxicity (COST) [12, 13]. Items were scored on a 0–4 scale and coded such that higher values reflected greater financial toxicity. Four items (assets, satisfaction, expenses, and control) were reverse‐coded prior to score calculation. The COST total score was computed as the sum of 11 items (range 0–44) and calculated only when all items were non‐missing. A dichotomous variable was coded for descriptive purposes using previously published COST cutoff scores (high financial toxicity: COST ≥ 22 [14, 15]; severe financial toxicity: COST ≥ 30 [16]).

#### 2.2.2 Health‐Related Quality of Life and Sleep Disturbance

HRQOL was assessed using the Functional Assessment of Cancer Therapy–General (FACT‐G) [17]. Outcomes included physical well‐being, social/family well‐being, emotional well‐being, functional well‐being, and the FACT‐G total score, with higher scores indicating better quality of life. Dichotomous variables for the FACT‐G total score and its subscales were created for descriptive purposes using ECOG performance status anchored cutoff scores, with low HRQOL defined as FACT‐G total ≤ 62, Physical Well‐Being ≤ 13, Functional Well‐Being ≤ 12, Emotional Well‐Being ≤ 16, and Social Well‐Being ≤ 20 [18].

Sleep disturbance was assessed using one item from the Patient Health Questionnaire (PHQ‐8), a screening tool for depression in adults: “Trouble falling or staying asleep, or sleeping too much”, scored from 0 (“not at all”) to 4 (“nearly every day”).

#### 2.2.3 Covariates

Multivariable models included demographic, socioeconomic, and clinical covariates based on past literature and clinical relevance. Covariates included: age in years (continuous), college education (yes/no), combined household income category (< $50K, ≥ $50–99 K, ≥ $100K, declined), partnered status (yes/no), time since diagnosis in years (continuous), treatment status (current treatment, hormone treatment, no active treatment), insurance type (private, state/public), cancer stage (categorical: Stage 0, Stage I, Stage II, Stage III, Stage IV), surgery type (lumpectomy, biopsy only, unilateral mastectomy, bilateral mastectomy), chemotherapy (yes/no), radiation therapy (yes/no), hormone therapy (yes/no), and body mass index in kg/m^2^ (continuous). Cancer stage was modeled categorically in the primary analyses to avoid potential confounding by metastatic disease.

### 2.3 Statistical Analysis Plan

Descriptive statistics were calculated for demographic, socioeconomic, clinical, financial toxicity, HRQOL, and sleep disturbance variables. Baseline characteristics were summarized overall and by combined household income category. Change in financial toxicity between baseline and 4‐month follow‐up was evaluated among participants with complete COST data at both time points using paired *t*‐tests, with Wilcoxon signed‐rank tests examined as a nonparametric sensitivity analysis. Longitudinal measurement invariance of the COST across baseline and follow‐up assessments was evaluated using confirmatory factor analysis with weighted least squares mean‐ and variance‐adjusted (WLSMV) estimation. Configural, metric, threshold, and strict invariance models were evaluated sequentially, with model comparisons based on relative fit indices (CFI, RMSEA, SRMR).

Adjusted baseline associations between baseline financial toxicity and baseline outcomes were estimated using separate multivariable linear regression models for each FACT‐G domain, the FACT‐G total score, and sleep disturbance. COST was modeled as a continuous predictor. Primary follow‐up analyses examined whether baseline financial toxicity predicted 4‐month follow‐up outcomes using separate multivariable linear regression models for each outcome. Follow‐up models adjusted for the corresponding baseline outcome in addition to all covariates listed above. Tests of significance were two‐sided (alpha = 0.05), and analyses were conducted in R. Sensitivity analyses tested reverse‐direction associations by estimating whether baseline HRQOL domains predicted follow‐up COST scores after adjustment for baseline COST and the same covariates.

## 3 Results

A total of 217 individuals consented to participate in the BOLD cohort; 182 completed the baseline survey and 146 completed both baseline and 4‐month follow‐up surveys. Baseline demographic, socioeconomic, and clinical characteristics are presented in Table 1. Participants were, on average, 57.1 years old (SD = 11.6) and a median of 2.5 years post‐diagnosis. Most were non‐Hispanic White (87%), college educated (74%), partnered (73%), and had private insurance (68%). Approximately one‐third had stage IV disease (36%). At baseline, 42 of 173 participants with complete COST data (24.3%) reported high or severe financial toxicity, including 22 participants (12.7%) with severe financial toxicity. Across HRQOL domains, the proportion below established impairment thresholds was highest for social/family well‐being (35.7% at baseline; 39.3% at follow‐up) and emotional well‐being (25.6% at baseline; 23.4% at follow‐up). The proportion below the FACT‐G total cutoff was 12.9% at baseline and 11.1% at follow‐up. Financial toxicity decreased modestly from baseline to follow‐up among participants with complete COST data at both time points (mean change = −1.06, 95% CI [−2.04, −0.08], *p* = 0.034). COST measurement models showed acceptable longitudinal fit across baseline and follow‐up assessments (Supporting Information S1: Table S1).

**TABLE 1:** Baseline demographic, socioeconomic, and clinical characteristics of the study sample, overall and by household income category.

Characteristic	*N*	Overall *N* = 182	Combined household income				*p*
			< $50K *N* = 27	≥ $50–99,999 *N* = 51	≥ $100K *N* = 75	Declined *N* = 16	
Age, yrs	182	57.10 (11.62)	57.59 (14.94)	57.69 (11.33)	55.17 (11.20)	63.31 (8.87)	0.037
Race/ethnicity	181						0.165
Hispanic		5 (2.8%)	0 (0%)	0 (0%)	3 (4%)	1 (6.7%)	
NH other		11 (6.1%)	4 (14.8%)	2 (3.9%)	4 (5.3%)	0 (0%)	
NH white		157 (86.7%)	23 (85.2%)	49 (96.1%)	68 (90.7%)	14 (93.3%)	
Unknown		8 (4.4%)	0 (0%)	0 (0%)	0 (0%)	0 (0%)	
College education	167						< 0.001
No		43 (25.7%)	14 (51.9%)	20 (39.2%)	6 (8.1%)	3 (20%)	
Yes		124 (74.3%)	13 (48.1%)	31 (60.8%)	68 (91.9%)	12 (80%)	
Partnered	168						< 0.001
0		45 (26.8%)	19 (73.1%)	16 (31.4%)	7 (9.3%)	3 (18.8%)	
1		123 (73.2%)	7 (26.9%)	35 (68.6%)	68 (90.7%)	13 (81.2%)	
BMI (kg/m^2^)	145	28.87 (6.70)	32.59 (9.51)	29.23 (6.42)	27.68 (5.71)	26.25 (4.44)	0.104
Years since dx	169	2.54	2.66	2.72	2.25	5.62	0.053
Cancer stage	181						0.865
0		5 (2.8%)	0 (0%)	2 (3.9%)	3 (4%)	0 (0%)	
1		54 (29.8%)	6 (23.1%)	13 (25.5%)	23 (30.7%)	7 (43.8%)	
2		39 (21.5%)	7 (26.9%)	10 (19.6%)	15 (20%)	3 (18.8%)	
3		17 (9.4%)	3 (11.5%)	4 (7.8%)	9 (12%)	0 (0%)	
4		66 (36.5%)	10 (38.5%)	22 (43.1%)	25 (33.3%)	6 (37.5%)	
Insurance type	168						< 0.001
Private		115 (68.5%)	6 (22.2%)	36 (70.6%)	63 (84%)	10 (66.7%)	
State		53 (31.5%)	21 (77.8%)	15 (29.4%)	12 (16%)	5 (33.3%)	
Surgery type	166						0.270
Bilateral mastectomy		41 (24.7%)	2 (9.1%)	13 (26.5%)	19 (27.5%)	4 (26.7%)	
Lumpectomy		63 (38%)	10 (45.5%)	14 (28.6%)	26 (37.7%)	9 (60%)	
Only biopsy		23 (13.9%)	3 (13.6%)	10 (20.4%)	7 (10.1%)	1 (6.7%)	
Unilateral mastectomy		39 (23.5%)	7 (31.8%)	12 (24.5%)	17 (24.6%)	1 (6.7%)	
Chemotherapy	177	97 (54.8%)	11 (42.3%)	31 (60.8%)	38 (52.8%)	8 (50%)	0.483
Radiation	177	104 (58.8%)	16 (61.5%)	33 (64.7%)	39 (54.2%)	10 (62.5%)	0.688
Hormonal therapy	177	136 (76.8%)	20 (76.9%)	34 (66.7%)	57 (79.2%)	15 (93.8%)	0.130

*Note:* Values are mean (SD), median, or *n* (%). Percentages were calculated among participants with non‐missing data for each characteristic. Household income category was based on combined household income and included participants who declined to report income as a separate category. *p* values compare characteristics across household income categories and were calculated using Kruskal‐Wallis tests for continuous variables and chi‐square tests with simulated *p* values for categorical variables.

Abbreviations: dx = diagnosis; NH = non‐Hispanic.

Table 2 displays adjusted associations between baseline financial toxicity and HRQOL and sleep disturbance at baseline and 4‐month follow‐up. At baseline, greater financial toxicity predicted lower physical well‐being (*B* = −0.271, *p* < 0.001), social/family well‐being (*B* = −0.188, *p* < 0.001), emotional well‐being (*B* = −0.206, *p* < 0.001), functional well‐being (*B* = −0.301, *p* < 0.001), and FACT‐G total score (*B* = −0.970, *p* < 0.001). Greater financial toxicity also predicted greater sleep disturbance at baseline (*B* = 0.051, *p* < 0.001).

**TABLE 2:** Associations between baseline financial toxicity (COST) and health‐related quality of life at baseline and 4‐month follow‐up (FU).

Outcome	*N*	*B*	SE	95% CI	*p*	Adj. *R* ^*2*^
Health‐related quality of life (baseline)						
Physical	124	−0.271	0.043	[−0.357, −0.185]	< 0.001	0.256
Social	124	−0.188	0.051	[−0.289, −0.087]	< 0.001	0.108
Emotional	122	−0.206	0.035	[−0.276, −0.137]	< 0.001	0.318
Functional	122	−0.301	0.052	[−0.404, −0.197]	< 0.001	0.252
Total	121	−0.97	0.136	[−1.240, −0.699]	< 0.001	0.346
Sleep disturbance	124	0.051	0.01	[0.032, 0.069]	< 0.001	0.186
Health‐related quality of life (4‐month FU)						
Physical	108	−0.078	0.037	[−0.151, −0.006]	0.035	0.624
Social	108	−0.07	0.034	[−0.137, −0.003]	0.039	0.63
Emotional	107	−0.071	0.031	[−0.131, −0.010]	0.024	0.576
Functional	106	−0.119	0.044	[−0.206, −0.032]	0.008	0.565
Total	105	−0.241	0.114	[−0.467, −0.014]	0.038	0.7
Sleep disturbance	105	0.001	0.01	[−0.019, 0.022]	0.890	0.24

*Note:* Higher COST scores indicate greater financial toxicity. Baseline models adjusted for age, college education, household income category, partnered status, time since diagnosis, treatment status, insurance type, cancer stage, surgery type, chemotherapy, radiation therapy, hormonal therapy, and body mass index. Four‐month FU models additionally adjusted for the corresponding baseline outcome.

Abbreviations: *B* = unstandardized regression coefficient for baseline COST score predicting the outcome; CI = confidence interval; COST = Comprehensive Score for financial Toxicity; FU = follow‐up; SE = standard error.

In follow‐up models adjusted for the corresponding baseline outcome and covariates, greater baseline financial toxicity predicted lower 4‐month physical well‐being (*B* = −0.078, *p* = 0.035), social/family well‐being (*B* = −0.070, *p* = 0.039), emotional well‐being (*B* = −0.071, *p* = 0.024), functional well‐being (*B* = −0.119, *p* = 0.008), and FACT‐G total score (*B* = −0.241, *p* = 0.038). Baseline financial toxicity did not predict 4‐month sleep disturbance (*B* = 0.001, *p* = 0.890). Reverse‐direction sensitivity analyses found no evidence that baseline HRQOL domains predicted follow‐up COST scores after adjustment for baseline COST and covariates (all *p* ≥ 0.14; Supporting Information S1: Table S2).

## 4 Discussion

Our results underscore the critical role of financial toxicity as both a concurrent and prospective correlate of HRQOL among breast cancer survivors. Although the cohort reported high average well‐being, a significant proportion experienced severe financial strain that was linked to poorer physical and psychosocial health. The finding that baseline financial toxicity was associated with lower follow‐up HRQOL across multiple domains, after adjustment for baseline HRQOL and clinical covariates including stage and time since diagnosis, suggests that financial distress is an important survivorship factor rather than merely a concurrent marker of poorer well‐being.

The pattern of baseline cross‐sectional results is consistent with prior studies linking greater financial toxicity to poorer quality of life across multiple domains among cancer survivors [1, 14, 19]. In our sample, survivors reporting greater financial toxicity concurrently reported lower well‐being across physical, social/family, emotional, and functional domains. In contrast, longitudinal associations between financial toxicity and follow‐up outcomes were smaller in magnitude but remained evident for physical, social/family, emotional, and functional well‐being, as well as FACT‐G total score. Thus, the pattern of results suggests that financial toxicity is associated with short‐term differences across multiple HRQOL domains, although not with follow‐up sleep disturbance after accounting for baseline sleep disturbance. The smaller longitudinal effect relative to cross‐sectional effects is consistent with prior prospective studies reporting modest longitudinal associations and heterogeneity across HRQOL domains [8, 9].

Because baseline financial toxicity was associated with follow‐up FACT‐G total score, the findings indicate that financial toxicity was related not only to domain‐specific HRQOL but also to overall HRQOL at follow‐up. In this sample, financial toxicity was associated with follow‐up physical, social/family, emotional, and functional well‐being. The persistence of the association with FACT‐G total score suggests that the domain‐specific associations were sufficiently consistent to be reflected in overall HRQOL. Similarly, the null longitudinal association with sleep disturbance may reflect the stability of the single‐item measure over time once baseline levels were covaried. However, longitudinal measurement invariance of the COST across baseline and follow‐up assessments was supported, indicating comparable measurement properties over time. This finding supports interpretation of the longitudinal models as reflecting associations between a consistently measured construct and subsequent differences in selected quality‐of‐life domains, rather than changes in item functioning or construct definition over time.

### 4.1 Implications

From a clinical perspective, these findings support consideration of routine assessment of financial toxicity in survivorship care. Greater financial toxicity was associated with lower subsequent physical, social/family, emotional, and functional well‐being and lower overall HRQOL, suggesting that screening may help identify survivors who may benefit from closer monitoring across multiple quality‐of‐life domains. Prospective intervention studies designed to evaluate both financial toxicity and domain‐specific quality‐of‐life outcomes are needed.

### 4.2 Limitations and Future Directions

Several limitations warrant consideration. First, the observational design precludes causal inference, and residual confounding and reverse temporal associations remain possible, although sensitivity analyses testing reverse‐direction associations were not significant (see Supporting Information S1: Table S2). Second, the 4‐month follow‐up interval may be insufficient to capture longer‐term associations between financial toxicity and outcomes that may change more slowly across survivorship. Third, the study focused on subjective financial toxicity measured by the COST and did not include objective indicators of financial hardship, which may show different longitudinal patterns. Finally, the analytic sample was, on average, relatively socioeconomically advantaged, with high rates of insurance coverage and educational attainment. Survivors experiencing more severe or persistent financial hardship may be less likely to enroll in or remain engaged in longitudinal survivorship research, which could attenuate observed change and limit generalizability. Future research would benefit from longer follow‐up periods, repeated assessments across multiple phases of survivorship, and integration of subjective and objective measures of financial hardship. Such designs would allow clearer characterization of how financial toxicity relates to changes in specific quality‐of‐life domains over time and across varying socioeconomic contexts.

## 5 Conclusion

Financial toxicity was associated with poorer HRQOL at baseline and with lower physical, social/family, emotional, functional, and overall HRQOL at follow‐up among breast cancer survivors. These findings support greater attention to financial toxicity in survivorship care and suggest that future longitudinal and intervention studies should evaluate domain‐specific QOL outcomes alongside financial hardship.

## Funding

This research was supported by the Masonic Cancer Center at the University of Minnesota by a National Institutes of Health National Cancer Institute grant (P30 CA77598), and by a grant from National Institute on Minority Health and Health Disparities (R00MD015296). Support for the use of REDCap was provided by a National Institutes of Health's National Center for Advancing Translational Sciences grant (UL1TR002494). The funders had no role in the implementation of this study or presentation of results.

## Ethics Statement

This cohort study was approved by the University of Minnesota Institutional Review Board (BOLD: STUDY00002747).

## Consent

All participants provided written informed consent.

## Conflicts of Interest

The authors declare no conflicts of interest.

## Supporting information


Supporting Information S1

## Data Availability

The data that support the findings of this study are available from the corresponding author upon reasonable request.

## References

[1] C. K. Altice , M. P. Banegas , R. D. Tucker‐Seeley , and K. R. Yabroff , “Financial Hardships Experienced by Cancer Survivors: A Systematic Review,” Journal of the National Cancer Institute 109, no. 2 (February 2017): djw205, 10.1093/jnci/djw205. PMC6075571 27754926

[2] S. S. Coughlin , J. X. Moore , and J. E. Cortes , “Addressing Financial Toxicity in Oncology Care,” Journal of Hospital Management and Health Policy 5doi (September 2021): 32, 10.21037/jhmhp-20-68. PMC8452270 34549166

[3] K. R. Yabroff , C. Bradley , and Y. T. Shih , “Understanding Financial Hardship Among Cancer Survivors in the United States: Strategies for Prevention and Mitigation,” Journal of Clinical Oncology 38, no. 4 (February 2020): 292–301, 10.1200/JCO.19.01564. PMC6994250 31804869

[4] H. M. Parsons , A. Gupta , P. Jewett , and R. I. Vogel , “The Intersecting Time, Administrative, and Financial Burdens of a Cancer Diagnosis,” Journal of the National Cancer Institute 117, no. 4 (April 2025): 595–600, 10.1093/jnci/djae252. PMC11972685 39392423

[5] G. L. Smith , M. P. Banegas , C. Acquati , et al., “Navigating Financial Toxicity in Patients With Cancer: A Multidisciplinary Management Approach,” CA: A Cancer Journal for Clinicians 72, no. 5 (September 2022): 437–453, 10.3322/caac.21730. PMC12994614 35584404

[6] T. T. Ngan , E. Tonorezos , M. Donnelly , and C. O'Neill , “Impact of Financial Toxicity on the Health‐Related Quality of Life and Financial Well‐Being of Cancer Patients and Survivors: A Comparative Study of the United Kingdom and United States,” Cancer Medicine 14, no. 2 (January 2025): e70606, 10.1002/cam4.70606. PMC11733585 39811923

[7] P. M. Carrera , H. M. Kantarjian , and V. S. Blinder , “The Financial Burden and Distress of Patients With Cancer: Understanding and Stepping‐Up Action on the Financial Toxicity of Cancer Treatment,” CA: A Cancer Journal for Clinicians 68, no. 2 (March 2018): 153–165, 10.3322/caac.21443. PMC6652174 29338071

[8] M. I. Liang , S. S. Summerlin , C. T. Blanchard , et al., “Measuring Financial Distress and Quality of Life Over Time in Patients With Gynecologic Cancer‐Making the Case to Screen Early in the Treatment Course,” JCO Oncology Practice 17, no. 10 (October 2021): e1576–e1583, 10.1200/op.20.00907. PMC8791820 33596114

[9] A. Wesevich , R. Dadwani , D. Hedeker , et al., “Longitudinal Associations Between Financial Toxicity and Health‐Related Quality of Life for Patients With Cancer,” JCO Oncology Practice 22, no. 4 (April 2026): 675–685, 10.1200/OP-24-00917. PMC13417836 40587814

[10] B. Yanez , L. M. Perry , J. D. Peipert , et al., “Exploring the Relationship Among Financial Hardship, Anxiety, and Depression in Patients With Cancer: A Longitudinal Study,” JCO Oncology Practice 20, no. 12 (December 2024): 1776–1783, 10.1200/OP.24.00025. PMC11649185 39146505

[11] P. I. Jewett , R. I. Vogel , P. Galchutt , et al., “Associations Between a Sense of Connection and Existential and Psychosocial Outcomes in Gynecologic and Breast Cancer Survivors,” Supportive Care in Cancer 30, no. 4 (April 2022): 3329–3336, 10.1007/s00520-021-06784-8. PMC8727470 34985561

[12] J. A. de Souza , B. J. Yap , K. Wroblewski , et al., “Measuring Financial Toxicity as a Clinically Relevant Patient‐Reported Outcome: The Validation of the Comprehensive Score for Financial Toxicity (COST),” Cancer 123, no. 3 (February 2017): 476–484, 10.1002/cncr.30369. PMC5298039 27716900

[13] J. A. de Souza , B. J. Yap , F. J. Hlubocky , et al., “The Development of a Financial Toxicity Patient‐Reported Outcome in Cancer: The COST Measure,” Cancer 120, no. 20 (October 2014): 3245–3253, 10.1002/cncr.28814. 24954526

[14] H. E. S. Ver , L. Ali‐Akbarian , S. N. Price , N. M. Lothfi , and H. A. Hamann , “Patient‐Reported Financial Toxicity, Quality of Life, and Health Behaviors in Insured US Cancer Survivors,” Supportive Care in Cancer 29, no. 1 (January 2021): 349–358, 10.1007/s00520-020-05468-z. PMC9208736 32361832

[15] S. Bouberhan , M. Shea , A. Kennedy , et al., “Financial Toxicity in Gynecologic Oncology,” Gynecologic Oncology 154, no. 1 (July 2019): 8–12, 10.1016/j.ygyno.2019.04.003. PMC7001853 31053404

[16] K. M. Esselen , A. Gompers , M. R. Hacker , et al., “Evaluating Meaningful Levels of Financial Toxicity in Gynecologic Cancers,” International Journal of Gynecological Cancer 31, no. 6 (June 2021): 801–806, 10.1136/ijgc-2021-002475. PMC9728957 33858954

[17] D. F. Cella , D. S. Tulsky , G. Gray , et al., “The Functional Assessment of Cancer Therapy Scale: Development and Validation of the General Measure,” Journal of Clinical Oncology 11, no. 3 (March 1993): 570–579, 10.1200/JCO.1993.11.3.570. 8445433

[18] T. Pearman , B. Yanez , J. Peipert , K. Wortman , J. Beaumont , and D. Cella , “Ambulatory Cancer and US General Population Reference Values and Cutoff Scores for the Functional Assessment of Cancer Therapy,” Cancer 120, no. 18 (September 2014): 2902–2909, 10.1002/cncr.28758. 24853866

[19] S. Pangestu and F. Rencz , “Comprehensive Score for Financial Toxicity and Health‐Related Quality of Life in Patients With Cancer and Survivors: A Systematic Review and Meta‐Analysis,” Value in Health 26, no. 2 (February 2023): 300–316, 10.1016/j.jval.2022.07.017. 36064514

