# Supplementary material for: Financial Toxicity and Follow‐Up Quality of Life Among Breast Cancer Survivors in the BOLD Cohort
Source: Psychooncology. 2026 Oct 14;35(10):e70630. doi: 10.1002/pon.70630 (PMC13663844; doi:10.1002/pon.70630)
Supplement: Supplementary file 1 [file PON-35-e70630-s001.docx]

**S1. Supplemental Methods: Longitudinal Measurement Invariance of COST**

Longitudinal measurement invariance of the 11-item Comprehensive Score for Financial Toxicity (COST) was evaluated across baseline (T1) and 4-month follow-up (T2). Analyses were conducted using confirmatory factor analysis in *lavaan* in *R* with a WLSMV estimator and theta parameterization given the ordinal indicators. All items loaded onto a single latent COST factor at each wave. Higher values reflected greater financial toxicity prior to modeling.

A sequence of nested multi-group models were tested with wave (T1 vs. T2) as the grouping variable: (1) configural invariance (same factor structure, no equality constraints), (2) metric invariance (equal factor loadings), (3) threshold invariance (equal loadings and thresholds; ordinal analogue of scalar invariance), and (4) strict invariance (equal loadings, thresholds, and residual variances). Model fit was based on changes in relative fit indices (ΔCFI, ΔRMSEA, ΔSRMR), and invariance was supported when |ΔCFI| ≤ .010, |ΔRMSEA| ≤ .015, and ΔSRMR ≤ .010.^20-22^

**Results**

Fit indices for all models are presented in Supplemental Table S1. The configural model demonstrated acceptable fit supporting the same one-factor structure across baseline and follow-up. Constraining factor loadings to equality (metric invariance) resulted in minimal change in model fit (ΔCFI = −0.001, ΔRMSEA = 0.002, ΔSRMR = 0.002). Adding equality constraints on item thresholds (threshold invariance) produced small changes in fit indices (ΔCFI = 0.002, ΔRMSEA = −0.021, ΔSRMR = −0.002). Finally, the strict invariance model (equal loadings, thresholds, and residual variances) also demonstrated minimal change relative to the preceding model (ΔCFI = −0.001, ΔRMSEA = 0.003, ΔSRMR = 0.002). Overall, results support longitudinal measurement invariance of the COST across baseline and 4-month follow-up, indicating that the latent construct and item functioning were stable over time in this cohort.

| **Supplemental Table 1 (S1).** Longitudinal measurement invariance of COST items (ordinal CFA; WLSMV; theta parameterization). | | | | | | | | |
| --- | --- | --- | --- | --- | --- | --- | --- | --- |
| **Step** | **CFI** | **TLI** | **RMSEA** | **90% CI** | **SRMR** | **ΔCFI** | **ΔRMSEA** | **ΔSRMR** |
| Configural | 0.996 | 0.995 | 0.070 | [0.052, 0.087] | 0.069 |  |  |  |
| Metric (loadings) | 0.995 | 0.994 | 0.072 | [0.055, 0.088] | 0.071 | -0.001 | 0.002 | 0.002 |
| Threshold (loadings + thresholds) | 0.997 | 0.997 | 0.051 | [0.031, 0.067] | 0.069 | 0.002 | -0.021 | -0.002 |
| Strict (add residuals) | 0.996 | 0.997 | 0.053 | [0.036, 0.069] | 0.071 | -0.001 | 0.003 | 0.002 |
| ***Note.*** Models are nested in the following order: configural, metric (loadings), threshold (loadings + thresholds), strict (loadings + thresholds + residuals). Δ indices are computed relative to the immediately preceding model. Decision rule shown uses \|ΔCFI\| ≤ .010, \|ΔRMSEA\| ≤ .015, and ΔSRMR ≤ .010. For WLSMV, chi-square difference tests may be unstable; Δ-fit indices are typically emphasized. | | | | | | | | |

**S2. Supplemental Methods: Reverse-Direction Sensitivity Analyses**

Sensitivity analyses were conducted to evaluate whether baseline health-related quality of life (HRQOL) and sleep were associated with subsequent financial toxicity. For each HRQOL exposure, a separate linear regression model regressed COST total score at 4-month follow-up on the corresponding baseline HRQOL domain (T1) and baseline COST. Exposures at baseline included FACT-G physical well-being, social/family well-being, emotional well-being, functional well-being, FACT-G total score, and sleep. All models adjusted for age at survey completion, college education, household income category, partnered status, time since diagnosis, treatment status, insurance at baseline, cancer stage, surgery, chemotherapy, radiation therapy, hormone therapy, and body mass index. Unstandardized regression coefficients *(B)*, standard errors *(SE)*, 95% confidence intervals *(CI)*, and *R²* values are reported.

**Results**

Across all models, baseline HRQOL and sleep were not associated with COST total score at 4-month follow-up after adjustment for baseline COST and covariates. Model R² values ranged from 0.762 to 0.767, reflecting substantial variance explained primarily by baseline COST and covariates. Overall, these findings do not support evidence of reverse-direction associations from baseline HRQOL or sleep to subsequent financial toxicity over the 4-month follow-up period.

| **Supplemental Table 2 (S2).** Sensitivity models predicting COST at 4-month follow-up (COST T2) from baseline outcomes (T1), adjusting for COST at baseline (T1) and covariates. | | | | | | |
| --- | --- | --- | --- | --- | --- | --- |
| **Model** | **B** | **SE** | **95% CI** | **p** | **Adj. R²** |  |
| Emotional well-being (T1) → COST (T2) | 0.179 | 0.194 | [-0.207, 0.566] | .359 | 0.711 |  |
| Functional well-being (T1) → COST (T2) | -0.139 | 0.124 | [-0.384, 0.107] | .265 | 0.713 |  |
| Physical well-being (T1) → COST (T2) | 0.050 | 0.145 | [-0.239, 0.340] | .729 | 0.711 |  |
| Social/family well-being (T1) → COST (T2) | -0.040 | 0.128 | [-0.294, 0.215] | .757 | 0.711 |  |
| FACT-G Total (T1) → COST (T2) | -0.023 | 0.077 | [-0.177, 0.131] | .769 | 0.707 |  |
| Sleep (T1) → COST (T2) | 0.166 | 0.681 | [-1.188, 1.520] | .808 | 0.711 |  |
| Note. Each model regressed COST total score at 4-month follow-up on the corresponding baseline outcome and baseline COST. All models additionally adjusted for age at survey randomization, college education, household income category, partnered status, metastatic disease status, time since diagnosis, treatment status, insurance at baseline, cancer stage, surgery, chemotherapy, radiation therapy, hormone therapy, and body mass index. B = unstandardized regression coefficient; CI = confidence interval. p values are two-sided. | | | | | | |
